# A Preliminary Comparative Assessment of the Role of CD8+ T Cells in Chronic Fatigue Syndrome/Myalgic Encephalomyelitis and Multiple Sclerosis

**DOI:** 10.1155/2016/9064529

**Published:** 2016-01-04

**Authors:** Ekua W. Brenu, Simon Broadley, Thao Nguyen, Samantha Johnston, Sandra Ramos, Don Staines, Sonya Marshall-Gradisnik

**Affiliations:** ^1^The National Centre for Neuroimmunology and Emerging Diseases, Griffith Health Institute, Griffith University, Gold Coast, Australia; ^2^School of Medicine, Griffith University, Gold Coast, Australia; ^3^School of Medical Science, Griffith University, Gold Coast, Australia

## Abstract

*Background*. CD8+ T cells have putative roles in the regulation of adaptive immune responses during infection. The purpose of this paper is to compare the status of CD8+ T cells in Multiple Sclerosis (MS) and Chronic Fatigue Syndrome/Myalgic Encephalomyelitis (CFS/ME).* Methods*. This preliminary investigation comprised 23 CFS/ME patients, 11 untreated MS patients, and 30 nonfatigued controls. Whole blood samples were collected from participants, stained with monoclonal antibodies, and analysed on the flow cytometer. Using the following CD markers, CD27 and CD45RA (CD45 exon isoform 4), CD8+ T cells were divided into naïve, central memory (CM), effector memory CD45RA− (EM), and effector memory CD45RA+ (EMRA) cells.* Results*. Surface expressions of BTLA, CD127, and CD49/CD29 were increased on subsets of CD8+ T cells from MS patients. In the CFS/ME patients CD127 was significantly decreased on all subsets of CD8+ T cells in comparison to the nonfatigued controls. PSGL-1 was significantly reduced in the CFS/ME patients in comparison to the nonfatigued controls.* Conclusions*. The results suggest significant deficits in the expression of receptors and adhesion molecules on subsets of CD8+ T cells in both MS and CFS/ME patients. These deficits reported may contribute to the pathogenesis of these diseases. However, larger sample size is warranted to confirm and support these encouraging preliminary findings.

## 1. Background

Chronic Fatigue Syndrome/Myalgic Encephalomyelitis (CFS/ME) is characterised by significant impairments in physical activity as a consequence of severe fatigue and other flu like symptoms. CFS/ME remains an unexplained disorder with substantial physiological impairments where diagnosis is based on self-report measures. Fatigue is present in other disorders, including Multiple Sclerosis (MS), where 70% of patients are plagued with fatigue and this can be disabling in a minority of patients [1–3]. This fatigue can be severe and has consequences on normal daily activity [4, 5]. CFS/ME has been reported in some MS patients where patients demonstrate classic CFS/ME symptoms such as intermittent headaches, malaise, and joint and muscle pain [6]. Nonetheless, diagnosis of MS also relies upon the demonstration of dissemination in time and space of central nervous system (CNS) lesions clinically or radiographically, often supported by biochemical and electrophysiological tests [7]. MS patients may be classified as relapsing remitting (RR-MS), primary-progressive (PP-MS), secondary-progressive (SP-MS), or clinically isolated syndrome [8].

MS is a neurological disorder characterised by inflammatory demyelination in the CNS while CFS/ME patients may experience nervous system manifestations of lack of concentration and autonomic symptoms [9–12]. Neuroimaging studies have suggested the presence of neuroinflammation in the midbrain of CFS/ME patients [11]. Despite some relative ambiguities in CFS/ME symptomatology, immune dysregulation is a common occurrence in CFS/ME as well as in MS. It has previously been postulated that symptom similarities between CFS/ME and MS may be explained by shared neuroimmune pathways [13]. The most common immune abnormality in both CFS/ME and MS is related to Natural Killer (NK) cell cytotoxic activity. Dysfunctional NK cell activity has been reported in patients with MS and CFS/ME. In MS patients, alterations in NK cell function and receptor expression contribute significantly to the pathogenesis of the disease [14, 15]. In CFS/ME, NK cell dysfunction involves decreased cytotoxic activity [16–18]. T cells have also been implicated in MS. Importantly, regulatory T cells (Tregs) are dysregulated and levels of these cells are related to the phase of the disease [19, 20].

Expansion of autoreactive lymphocytes in MS results in inflammatory and active immune responses in the CNS as these lymphocytes are able to migrate to the CNS and induce damage [21]. Reducing the activities of these autoreactive lymphocytes is the fundamental goal of many therapeutic interventions in MS [22]. CD8+ T cells have been extensively studied in MS owing to their presence in the CNS lesions of MS patients. In MS, pathogenic CD8+ T cells may induce proinflammatory reactions via interleukin- (IL-) 17 and interferon- (IFN-) *γ* and eliminate oligodendrocytes while regulatory CD8+ T cells suppress autoreactive CD4+ T cells reactions and promote anti-inflammatory reactions [23]. In CFS/ME patients CD8+ T cells may display diminished levels of activation, reduced cytotoxicity, and low numbers of effector memory cells [18, 24].

It is apparent that CD8+ T cells are involved in the pathogenesis of CFS/ME and MS; hence, the aim of this study was to determine whether dysregulation in cytotoxic CD8+ T cells follows a similar pattern in CFS/ME and MS.

## 2. Methods

### 2.1. Subjects

CFS/ME participants were defined according to the International Consensus Criteria (ICC) [25]. Disability in the CFS/ME patients was measured using Dr. Bell's Disability Adjustment scale [26]. MS cases were clinically diagnosed as having MS according to the revised McDonald criteria [7]. MS disease progression and responsiveness were assessed using the Expanded Disability Status Scale (EDSS) [27] and disease severity was measured using the MS Severity Scale (MSSS) [7]. Nonfatigued controls had no incidence of CFS/ME or MS and were in good health without evidence of fatigue. Excluded from the study were smokers, pregnant woman, breastfeeding, or having been clinically diagnosed with any other major diseases. All subjects gave informed written consent to participate in the study and the study received ethical approval from the Griffith University Human Ethics Committee (MSC/18/13/HREC) prior to commencement.

### 2.2. Assessment of CD8+ T Cell Phenotypes

Whole blood (10 mL) was collected from all participants and analysed within 12 hours of collection. To identify subsets of CD8+ T cells at different stages of differentiation, samples were labelled with fluorochrome conjugated monoclonal antibodies, including CD3, CD8, CD27, and CD45RA (CD45 exon isoform 4). Cells were analysed on the Fortessa 2.0 (Becton Dickenson (BD) Biosciences, San Jose). For each CD8+ T cell assessment, forward and side scatter plots were used to determine the lymphocyte population. Cells of interest were identified from the lymphocyte population as cells expressing CD3+ and CD8+. The expression of cytokines, chemokine receptors, adhesion molecules, and migratory molecules on CD8+ T cells were also examined using the following markers: CCR5, CCR7, CXCR3, CD49d, CD29, CD18, CD11a, PSGL-1, and CD127. Glycoprotein, CD44, was also examined.

### 2.3. Assessment of CD8+ T Cell Receptors

Inhibitory receptors were measured in whole blood cells stained with monoclonal antibodies including KLRG1, LAG3, CTLA4, and BTLA. The expression patterns of these inhibitory receptors were examined on the CD8+ T cell phenotypes. Coexpression of these receptors was also assessed on subsets of CD8+ T cells.

### 2.4. Statistical Analysis

Statistical analyses were executed using SPSS (version 18.0, SPSS Inc., Chicago, USA) and Graph Pad Prism (version 6.0, Graph Pad Software, Inc., San Diego, USA). A test for normality was performed using the Kolmogorov-Smirnov tests. ANOVA was used to determine significance for normally distributed data while the independent sample Kruskal Wallis test was used as the nonparametric. Bonferroni analysis was used to assess significant parameter differences post hoc. Pearson chi square test was used to determine significant gender differences. *P* values less than or equal to 0.05 were considered significant. The data is expressed as either median or mean ± standard error of the mean (SEM).

## 3. Results

### 3.1. Subject Characteristics

The characteristics of the participants recruited in the study are outlined in Table 1. A number of the CFS/ME patients were taking a combination of different medications at the time of the study. These medications include anticholinergic (*n* = 1), antihistamine (*n* = 1), antidepressant (*n* = 10), blood pressure medication (*n* = 1), steroids (*n* = 2), anticonvulsants (*n* = 4), Benodiazepines (*n* = 1), opioid receptor antagonist (*n* = 1), asthma (*n* = 3), cardiotonic agent (*n* = 2), anti-inflammatory (*n* = 3), opioids (*n* = 2), opioid analgesics (*n* = 4), triptans (*n* = 1), proton pump inhibitors (*n* = 3), vitamins and supplements (*n* = 5), anticoagulants (*n* = 2), and laxatives (*n* = 1). Nine of the CFS/ME patients were on no medications at the time of the study. Mean disability in the CFS/ME cases was 47.14%  ± 2.20 (SD) using Dr. Bell's Disability score and classifying CFS/ME as moderate CFS/ME patients as described [28] (Table 2).

MS patients were not on any immunomodulatory therapies during this study, nor had they taken these previously. Of the 11 MS patients, there were relapsing-remitting (*n* = 4), secondary-progressive (*n* = 2), primary-progressive (*n* = 2), and clinically isolated syndrome (*n* = 3) cases. The average number of relapses (ever) rate among the MS cases was 2.4 ± 0.55. MS mean age was 56.0 ± 4.9 (SD) years reported illness onset of an average of 6.11 years ± 2.45 (SD) for a duration of 13.76 years ± 3.83 (SD). The mean EDSS and MSSS scores for the MS patients were 2.41 ± 0.79 (SD) and 2.85 ± 0.89 (SD), respectively, which classifies MS patients as moderately disabled (Table 2).

In each group there were a large percentage of females in comparison to males but there was no significant difference in gender. Full blood count analyses were performed on all samples to determine the distribution of the different blood cells (Table 1). White blood cells, lymphocytes, monocytes, and eosinophils were significantly higher in the MS group compared to the other groups. CD8+ T cell phenotypes CD27 and CD45RA surface markers were used to determine lineage differentiation (naïve and memory phenotypes) of the CD8+ T cells. Four different subsets of CD8+ T cells were characterized including CD8+CD3+CD27+CD45RA+ cells (naïve), CD8+CD3+CD27+CD45RA− cells (central memory [2]), CD8+CD3+CD27−CD45RA− cells (effector memory [EM]), and CD8+CD3+CD27−CD45RA+ cells (CD45RA+ effector memory [EMRA]). There were no significant differences in total CD8+ T cells, naïve, CM, EM, and EMRA CD8+ T cells among the three groups (Table 3).

#### 3.1.1. Quantitation of Inhibitory Receptors on CD8+ T Cells

**Figure 1:**
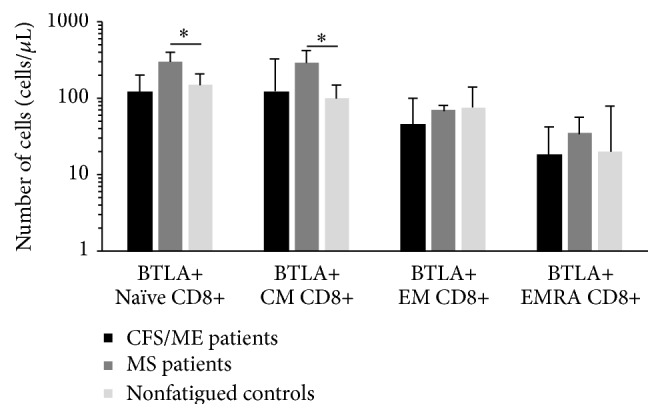
Expression of BTLA on CD8+ T cells in CFS/ME, MS, and nonfatigued controls. BTLA was increased on naïve and CM CD8+ T cells in the MS compared with controls. Data is represented as median ± SEM, where *∗* represents *P* < 0.05 (EM: effector memory, EMRA: effector memory RA, and CM: central memory).

Surface expression of the following inhibitory receptors was examined on the CD8+ T cells, KLRG1, LAG3, CTLA4, and BTLA. These receptors were measured on total CD8+ T cells and subsets of CD8+ T cells at different stages of differentiation as previously described. Only BTLA was significantly elevated in the naïve and CM CD8+ T cells from the MS patients compared with the CFS/ME patients and the nonfatigued controls (Figure 1).

#### 3.1.2. Expression Pattern of Cytokine and Chemokine Receptors

**Figure 2:**
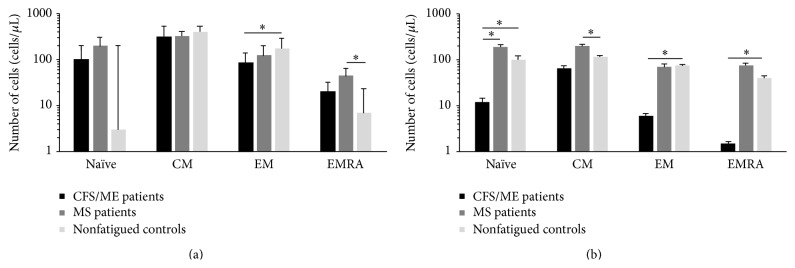
Expression of receptors in CFS/ME, MS, and nonfatigued controls. (a) CD49d/CD29 was reduced in EM subsets of CD8+ T cells in the CFS/ME patients but elevated in EMRA subsets of CD8+ T cells in MS patients compared to controls. (b) CD127 expression was reduced on naïve, EM, and EMRA subsets of CD8+ T cells in the CFS/ME patients but not CM subsets as CD127+ CD8+ T cells were evaluated in MS patients compared with controls. MS patients also demonstrated elevated naïve CD8 T cells compared with controls. Data is represented as median ± SEM, where *∗* represents *P* < 0.05 (EM: effector memory, EMRA: effector memory RA, and CM: central memory).

**Figure 3:**
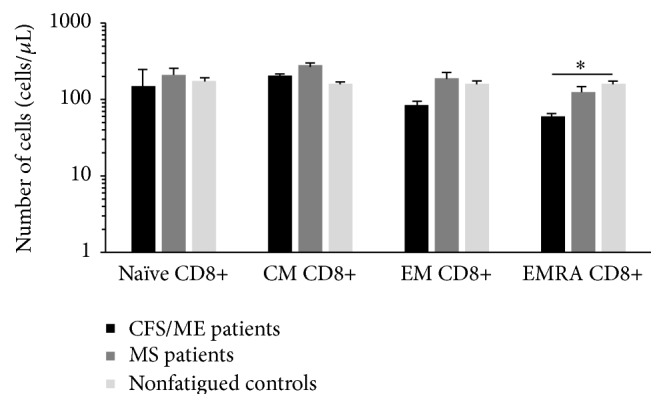
Expression of adhesion molecules on subsets of CD8+ T cells in CFS/ME, MS, and nonfatigued controls. Expression levels of PSGL-1 were reduced on EMRA CD8+ T cells in the CFS/ME patients compared with the nonfatigued controls. Data is represented as ± SEM, where *∗* represents *P* < 0.05 (EM: effector memory, EMRA: effector memory RA, and CM: central memory).

Expression of cytokine receptors including CCR7, CCR5, and CD127 was measured on total CD8+ T cells and subsets of CD8+ T cells at different stages of differentiation. CD49/CD29 was significantly reduced on the EM CD8+ T cells of the CFS/ME patients in comparison to the nonfatigued controls, while in the MS patients, CD49d/CD29 was significantly elevated in the naïve and EMRA CD8+ T cells in comparison to the nonfatigued controls (Figure 2(a)). When the expressions of these receptors were examined, significant decrease in the expression of CD127+ was observed on most subsets of CD8+ T cells from the CFS/ME patients while in the MS patients, CD127 expression was reduced on naïve, EM, and EMRA subsets of CD8+ T cells in the CFS/ME patients (Figure 2(b)). Differential levels of integrins and selectins and cell surface glycoproteins PSGL, KLRG1, CD11a/CD18, and CD44 were measured on total and subsets of CD8+ T cells at different stages of differentiation. PSGL-1 was significantly reduced on EMRA CD8+ T cells in the CFS/ME patients in comparison to the nonfatigued controls (Figure 3).

## 4. Discussion

This preliminary study has identified significant impairments in subsets of CD8+ T cells in CFS/ME and MS patients. Overall the MS patients showed significant differences in the expression of receptors and adhesion molecules in comparison to the CFS/ME patients. These results demonstrate CD8+ T cells might play a role in the pathogenesis of MS compared with CFS/ME. PSGL-1 is elevated on CD4+ T cells in RR-MS patients and this may suggest an important role in the transmigration of lymphocytes to the CNS [29]. However, total CD8+ T cells in these patients have stable levels of PSGL-1 [29]. Therefore, the expression of PSGL-1 on CD8+ T cells may to some extent be dependent on the stage and type of disease. Nonetheless, it is possible that these cells have high affinity to transmigrate the blood brain barrier (BBB) and this may be specific to naïve CD8+ T cells in MS. In MS, PSGL-1 is important for the adhesion and recruitment of CD8+ T cells to the inflamed CNS [30]. CD49d/CD29 represents the *α*4*β*1 integrin, adhesion molecules which are important during inflammation. CD49d/CD29 has been shown to be significantly elevated on PBMCs in demyelinated lesions in the CNS [31]. An increase in the expression of *α*4*β*1 on the cell surface may suggest increased migration of CD8+ T cells to the CNS. High levels of *α*4*β*1 on the CD8+ T cells in particular, in the naïve and EMRA subsets may indicate a reduced prevalence of soluble VCAM-1, IFN-*γ*, and TNF [32]. Soluble VCAM-1 is known to suppress the function of *α*4*β*1 under normal physiological concentration, while in the CFS/ME patients decreased expression of CD49d/CD29 on EM CD8+ T cells may indicate reduced migration of effector cells to sites of inflammation. CD127 is the receptor for IL-7 and is an important marker for T cell maturation and function. Binding of IL-7 to this receptor is a vital component in the release of granzymes resulting in demyelination [33]. In MS polymorphism in the CD127 gene sequence is an associated risk factor [34]. CD127 has four different haplotypes. Haplotype 1 results in the production of enormous amounts of sCD127; haplotype 2 is associated with a less soluble form of sCD127 due to low levels of exon 6 splicing and is associated with a lower risk of MS [35]. Homozygosity for haplotype 4 increases the likelihood of MS [36]. It has been suggested that polymorphisms in CD127 and its cytokine IL-7 may be correlated with susceptibility to MS [37]. The MS group in this study demonstrated heightened levels of CD127+ on all subsets of CD8+ T cells in comparison to the CFS/ME patients but this was only significant in the EMRA CD8+ T cells. Interestingly, IL-7 may inhibit the function of VCAM-1 by binding to it and thus allowing the dominance of *α*4*β*1 integrin which facilitates the movement of T cells to the CNS. In the CNS, CD8+ T cell cytotoxic activity may be further enhanced by IL-7 resulting in an overabundance of granzymes and consequently increasing demyelination in the CNS [38, 39]. The expression of CD127+ was decreased on most CD8+ T cell subsets in the CFS/ME patients compared with both MS and controls. Reduced CD127 in the CFS/ME patients was corroborated with reduced *α*4*β*1 integrin confirming significant alterations in the migratory potential of the CD8+ T cells in CFS/ME patients. The exact role of CD127 on CD8+ T cells in CFS/ME is unclear though it has been suggested that reduced CD127 on exhausted CD8+ T cells might be responsible for the inability for CD8+ T cells to suppress viral persistence [40]. CD8+ T cell exhaustion has been previously suggested in CFS/ME owing to the overwhelming levels of other exhaustion markers including PD1 and CD95 [24]. BTLA is another inhibitory coreceptor expressed on CD8+ T cell; similar to CD127, BTLA expression was increased in the MS patients compared to the CFS/ME patients. BTLA is known to bind to TNF receptor family member herpesvirus entry mediator (HVEM). This initiates a sequence of events involving phosphorylation of ITIM motifs and induction of phosphatases SHP-1 and SHP-2 [41]. BTLA and HVEM interact in either cis or trans configuration and this inhibits or activates NF-*κ*B, respectively [42]. Increased expression of BTLA on naïve and CM CD8+ T cells may indicate suppression of T cell receptor signalling via CD3 and/or CD28 [43]. Additionally, BTLA interaction with HVEM promotes cell survival and memory generation of effector CD8+ T cells [44].

Previous research has indicated decreases in total CD8+ T cells in particularly EM and EMRA CD8+ T cells [45, 46]. However, in the present study although total CD8+ EM and EMRA T cells were reduced in MS patients this was not statistically significant.

## 5. Conclusions

In summary, these preliminary findings provide new insight into the possibility of hyper activated inflammatory CD8+ T cell profile in untreated MS patients while CFS/ME patients may display an exhausted profile which permits viral prevalence and persistence. The above data may suggest that the differential expressions of receptors and adhesion molecules in MS patients are in response to imbalances in neuroimmune homeostasis. In comparison to CFS/ME patients, MS patients may have more severe immune dysregulation. Nevertheless it is likely that impairments in CD8+ T cells in CFS/ME patients relate to abnormal levels of adhesion and migratory molecules and these abnormalities may contribute to the persistent immune dysregulation observed and warrant further validation in a larger sample size.

## Figures and Tables

**Table 1:** Characteristics of participants and blood parameters.

	CFS/ME	MS	Controls	*P* value			
				Overall	MS versus CFS/ME	MS versus control	CFS/ME versus control
Participants (*n*)	23	11	30				
Age (years)	49.0 ± 2.5	56.0 ± 4.9	53.5 ± 2.2	0.72	>0.99	>0.99	>0.99
Females, *n* (%)	17 (73.9)	10 (90.9)	19 (63.3)	0.44			
Haemoglobin (g/L)	135.0 ± 2.25	136.0 ± 2.90	139.0 ± 2.35	0.24	>0.99	0.89	0.32
White cell count (×10^9^/L)	5.60 ± 0.32	6.80 ± 0.64	6.00 ± 0.25	0.04^*∗*^	0.04^*∗*^	0.52	0.28
Platelets	228.0 ± 13.34	264.0 ± 15.80	249.50 ± 10.69	0.76	>0.99	>0.99	>0.99
Haematocrit (%)	0.41 ± 0.01	0.40 ± 0.01	0.41 ± 0.01	0.35	>0.99	>0.99	0.49
Red cell count (×10^12^/L)	4.49 ± 0.07	4.55 ± 0.13	4.61 ± 0.07	0.53	>0.99	>0.99	0.79
MCV (fL)	89.17 ± 0.69	89.20 ± 0.85	89.53 ± 0.68	0.72	>0.99	>0.99	>0.99
Neutrophils (×10^9^/L)	3.38 ± 0.25	4.20 ± 0.49	3.53 ± 0.17	0.39	0.53	0.82	>0.99
Lymphocytes (×10^9^/L)	1.63 ± 0.09	2.30 ± 0.23	1.95 ± 0.10	0.001^*∗*^	0.001^*∗*^	0.13	0.11
Monocytes (×10^9^/L)	0.31 ± 0.02	0.45 ± 0.03	0.33 ± 0.02	0.0004^*∗*^	0.002^*∗*^	0.0004^*∗*^	>0.99
Eosinophils (×10^9^/L)	0.14 ± 0.015	0.21 ± 0.03	0.12 ± 0.01	0.02^*∗*^	0.13	0.01^*∗*^	0.91
Basophils (×10^9^/L)	0.02 ± 0.004	0.03 ± 0.004	0.02 ± 0.003	0.41	0.56	>0.99	>0.99
ESR (mm/Hr)	10.50 ± 2.59	13.00 ± 5.29	10.00 ± 1.91	0.21	>0.99	0.89	0.26

Data is represented as mean ± SEM, where *∗* represents *P* < 0.05.

**Table 2:** Clinical characteristics of CFS/ME and MS.

	CFS/ME (*n* = 23, mean ± SD)	MS (*n* = 11, mean ± SD)
Dr. Bell's Disability	47.14% ± 2.20	
Expanded Disability Status Scale		2.41 ± 0.79
Multiple Sclerosis Severity Scale		2.85 ± 0.89
Courses		
(i) Relapsing-remitting		*n* = 4
(ii) Secondary-progressive		*n* = 2
(iii) Primary-progressive		*n* = 2
(iv) Clinically isolated syndrome		*n* = 3
Age of onset (years)	35.28 ± 4.63	6.11 ± 2.45
Duration (years)	14.96 ± 8.87	13.76 ± 3.83
Relapses rate		2.4 ± 0.55

**Table 3:** Distribution of total and subsets of CD8+ T cells in CFS/ME patients, MS patients, and nonfatigued controls.

CD8+ T cells (%)	CFS/ME	MS	Nonfatigued controls	*P* value			
				Overall	MS versus CFS/ME	CFS/ME versus control	MS versus control
Total CD8+ T cells	13.25 ± 0.86	12.36 ± 1.89	13.24 ± 0.88	0.84	>0.99	>0.99	>0.99
Naïve CD8+ T cells	33.86 ± 3.89	36.76 ± 5.76	24.20 ± 2.19	0.07	>0.99	0.12	0.29
CM CD8+ T cells	31.88 ± 2.63	38.50 ± 2.36	32.93 ± 2.75	0.51	0.81	>0.99	0.88
EM CD8+ T cells	12.52 ± 2.15	15.83 ± 2.77	18.64 ± 2.13	0.07	>0.99	0.08	0.51
EMRA CD8+ T cells	10.18 ± 2.21	6.86 ± 2.22	13.38 ± 2.13	0.06	0.82	0.43	0.08

## Abbreviations

CD: Cluster of differentiation
MS: Multiple Sclerosis
CFS/ME: Chronic Fatigue Syndrome/Myalgic Encephalomyelitis
CM: Central memory
EM: Effector memory
EMRA: Effector memory cells expressing CD45RA
BTLA: B- and T-lymphocyte attenuator
KLRG1: Killer cell lectin-like receptor subfamily G member 1
LAG3: Lymphocyte-activation protein 3
CTLA-4: Cytotoxic T-lymphocyte-associated protein 4
CCR7: Chemokine receptor type 7
CCR5: Chemokine receptor type 5
VCAM-1: Vascular cell adhesion protein 1
IFN-*γ*: Interferon gamma
TNF: Tumor necrosis factor
IL-7: Interleukin-7
CNS: Central Nervous System
PD1: Programmed cell death protein 1
HVEM: Herpes virus entry mediator
NF-*κ*B: Nuclear factor kappa-light-chain enhancer of activated B cells.
